# Current State of Treatment for Alcohol and Other Drug Use Disorders in Adolescents

**Published:** 2009

**Authors:** Deborah Deas, Andrew Clark

**Keywords:** Underage drinking, adolescent, alcohol and other drug use disorders, treatment method, psychosocial treatment method, treatment outcome, literature review

## Abstract

Over the past he decade, treatment programs have been developed specifically for adolescents with alcohol and other drug (AOD) use disorders. The vast majority of these programs use psychosocial approaches, which can be further classified into family-based interventions and multisystemic therapy, motivational enhancement therapy, behavioral therapy, and cognitive–behavioral therapy. Outcome studies have evaluated the effectiveness of the different approaches. The results indicate that all of these strategies can improve an adolescent’s outcome on a variety of measures. Pharmacotherapy rarely is used in the treatment of adolescents with AOD use disorders, and existing studies only have assessed the effectiveness of agents aimed at treating coexisting psychiatric conditions. Future studies should use more consistent, state-of-the-art assessment instruments developed specifically for adolescents and also pay greater attention to an adolescent’s developmental status and its impact on treatment outcome.

Over the past decade, researchers and clinicians have made substantial progress in treating adolescents with alcohol and other drug (AOD) use disorders, including developing treatment programs specifically for this age-group rather than administering the same treatments as for adults with these disorders. And although the knowledge base regarding what constitutes appropriate treatment for adolescents still remains sparse and in need of improvement, as described in the accompanying article by Wagner (pp. 67–75), researchers and clinicians now are recognizing that adolescents differ from adults and have specific developmental characteristics which may influence treatment design, patient adherence to treatment, and treatment outcome (Deas et al. 2000*b*). As a result, treatment approaches have been developed to specifically target adolescents with AOD use problems, and their success has been evaluated in outcome studies. This article reviews the findings of these outcome studies.

## Inclusion Criteria

Although many approaches—primarily psychosocial strategies—have been used to treat adolescents with AOD use problems, the effectiveness of most of these treatments has not been evaluated in clinical trials that meet usual scientific standards (i.e., studies conducted under controlled conditions that compare the outcomes of two or more treatments). To identify those studies that do meet the criteria for controlled, comparative trials, an extensive literature search was conducted (for more information, see Deas 2008). The reference lists of each of those studies also were reviewed to identify additional studies not captured in the initial literature search.

To be included in the review, the studies had to meet the following criteria:

- They had to focus on AOD use disorders in adolescents;
- They had to compare one or more treatment modalities;
- They had to be published between 1990 and 2005; and
- They had to involve random assignment of participants to the different treatment conditions.

The literature search identified 14 studies that met these criteria and which fell into five categories of treatment strategies:

- Family-based interventions and multisystemic therapy;
- Motivational enhancement therapy;
- Behavioral therapy;
- Cognitive–behavioral therapy (CBT); and
- Pharmacotherapy.

The following sections review the main characteristics and findings of these studies, thereby providing an overview of currently available, evidence-based treatments. In addition to showing true promise for treating adolescents, these approaches may be useful in other settings and should help to inform future research efforts.

## Family-Based Interventions and Multisystemic Therapy

The most thoroughly investigated type of adolescent AOD treatment involves family-based interventions that are conducted in the office or home environment and require specific training. These approaches are based on the assumption that people’s behaviors are heavily influenced by the context in which they experience their primary relationships—which typically is the family. Of course, other contexts, such as the social environment (i.e., peer groups) and community, also influence behavior in adolescents, but the primary influence in this age-group is thought to be the family. Therefore, both assessment and treatment of AOD use in adolescents should take into consideration the adolescent’s functioning within the family as well as his or her interactions with the extended family and social systems. The most studied evidence-based treatment taking this broader ecological approach in the natural environment is known as multisystemic therapy (MST).

Overall, the literature search identified seven studies that assessed the effectiveness of family-based and MST approaches, as reviewed below.

### Study by Lewis and Colleagues (1990)

This study included 84 adolescents (ages 12–20, with a mean age of 16 years) who were randomly assigned to 12 weeks of either family therapy (i.e., a program called the Purdue Brief Family Therapy Program) or a family education intervention (i.e., a program called Training in Parenting Skills). To reduce adolescent AOD use, the Purdue Brief Family Therapy Program combines a variety of strategies aimed at achieving the following:

- Acknowledging that AOD use is a problem not only of the adolescent but of the entire family;
- Reducing the family’s resistance to AOD treatment;
- Reestablishing appropriate parental influence and interrupting dysfunctional family behaviors;
- Assessing how AOD use affects relationships among family members;
- Implementing change strategies; and
- Helping the adolescent and his or her siblings resist peer pressure to use AODs by enhancing assertion skills.

In contrast, the Training in Parenting Skills program educated all family members about different types of drugs (i.e., “soft” drugs, such as tobacco, alcohol, and marijuana, and “hard” drugs, which include all other illegal drugs) and their effects and provided information on ways to overcome AOD addiction. Treatment outcome was assessed by determining frequency of AOD use and a “drug severity index.”

The study found that adolescents in the family therapy group were more likely to have a lower drug severity index after treatment than those in the family education group; moreover, adolescents in the family therapy group exhibited greater overall improvement throughout the treatment period.

### Study by Henggeler and Colleagues (1991)

This study included 200 adolescents (mean age 14.4 years) who were involved in the juvenile justice system for a variety of offenses (e.g., truancy and other crimes), including 26 adolescents with AOD-related offenses (e.g., public intoxication or drug possession or sale). The study comprised two parts. In the first part, the investigators randomly assigned the participants to MST or individual counseling. The MST modality was a time-intensive, individualized approach that focused on changing the adolescents’ behavior in a natural environment. To this end, the investigators identified the strengths and weaknesses of each participant and developed a treatment plan based on these to facilitate change. For example, for an adolescent who was an excellent basketball player but made poor decisions with respect to choosing deviant peers, a treatment plan would focus on increasing his pro-social basketball opportunities with non–substance-using peers and teaching him improved peer refusal skills. Treatment involved therapy sessions in the adolescent’s home as well as information for the parents on how to handle difficult situations during the treatment period. The individual-counseling approach, in contrast, targeted personal, family, and academic issues and focused solely on the adolescent, ignoring the multiple systems to which he or she was connected.

Treatment outcome in this part of the study was determined based on the rate of repeated AOD-related arrests rather than actual quantity and frequency of AOD use. Although treatment duration was comparable (i.e., mean of 24 hours for MST versus 28 hours for individual counseling), adolescents receiving MST had significantly lower AOD-related arrest rates during the study period compared with adolescents receiving individual counseling (i.e., 4 percent and 16 percent, respectively).

For the second part of the study, the investigators compared the effects of MST and the Department of Youth Services’ usual program for juvenile offenses and AOD use. In that program, adolescents had monthly meetings with a probation officer to evaluate school attendance and compliance with curfews. Both interventions were delivered over a 4-month period. The frequency of use of soft drugs (i.e., alcohol and marijuana) and hard drugs (i.e., cocaine, hallucinogens, amphetamines, barbiturates, and heroin) was measured to determine outcome. The study found that adolescents in the MST group had significantly lower alcohol and marijuana use rates compared with the adolescents in the Department of Youth Services’ usual program.^1^

### Study by Henggeler and Colleagues (1999)

The same investigators conducted another study involving 118 juvenile offenders (ages 12–17 years, mean age 15.7 years) who were randomly assigned to MST or a program offered by the community. Adolescents assigned to MST received an average of 130 days of treatment that included about 40 hours of direct contact with a treatment provider and 6 hours of indirect contact. The program offered by the community involved outpatient substance abuse treatment and weekly meetings in a 12-step program for an average of 5 months but included few specialized substance abuse or mental health services. The main outcome assessed was AOD use as determined by a questionnaire and urine screens.

The study found that at the end of treatment, adolescents in the MST group used significantly less alcohol, marijuana, and other drugs. Moreover, 6 months after the end of treatment, adolescents in the MST group appeared to have fewer overall problems, as indicated by fewer placements outside the home (e.g., in juvenile detention facilities, resident treatment facilities, or other institutions) compared with adolescents in the community program. Finally, the effects of treatment appeared to persist over an extended period of time because 4 years later, a followup of 80 participants (Henggeler et al. 2002) showed that participants in the MST group had fewer convictions for aggressive criminal behaviors and higher levels of abstinence from marijuana than participants in the community program.

### Study by Joanning and Colleagues (1992)

This investigation compared three different psychosocial treatment approaches for adolescent AOD use in 134 adolescents (ages 11–20):

- Brief strategic family therapy that involved structural components (i.e., establishing a hierarchy and boundaries) as well as strategic components (i.e., specific problem-focused interventions)^2^ and which was provided in 7 to 15 therapy sessions delivered weekly for 12 weeks;
- Adolescent group therapy (delivered weekly for 12 weeks) that was similar to outpatient group therapy offered by hospitals and which sought to enhance social skills, cognitive development, and role playing; or
- Family drug education, which provided information about drug use and its effects on both the adolescent and his or her family in a setting of several families and which was provided biweekly for six sessions.

The main outcome measured was AOD use, which was determined indirectly through random drug screens, legal involvement, drug involvement surveys, and family assessment interviews. Direct assessment of quantity and frequency of AOD use was not performed.

The study found that after treatment, adolescents in the family systems therapy group were significantly more likely to be abstainers (54 percent) than those in the adolescent group therapy (28 percent) or family drug education group (16 percent). No differences were found among the three groups in measures of family functioning.

### Study by Latimer and Colleagues (2003)

Another group of researchers compared the effectiveness of family therapy combined with CBT versus a drug harm psycho-education curriculum in 43 adolescents (ages 12–18, with a mean age of 16.07 years). The family therapy/CBT approach promoted abstinence from drugs by strengthening family communication, age-appropriate roles, and effective parenting skills. It also used behavioral contracts to encourage desired behaviors. The cognitive–behavioral component focused on helping the adolescents understand the principles of problem solving and of rational versus emotive behaviors. The drug harm psycho-education curriculum, which was based on drug information from the National Institute on Drug Abuse, provided information on the harmful effects of AODs and the negative consequences associated with their use.

Both interventions were delivered for 16 weeks, and the researchers conducted follow-up evaluations at 1, 3, and 6 months after treatment, measuring AOD use frequency, presence of drugs in the urine, and self-report interviews for all types of drugs. The investigators found that adolescents receiving the family therapy/CBT intervention on average had fewer days of alcohol use during the 6-month follow-up period and had lower marijuana use rates at 6 months compared with adolescents who had received the drug harm psycho-education intervention.

### Study by Waldron and Colleagues (2001)

These investigators compared the outcomes of 114 AOD-abusing adolescents (ages 13–17, with a mean age of 15.4 years) who were randomly assigned to one of four interventions:

- Functional family therapy, which included 12 hours of therapy (one session per week);
- CBT, which included 12 hours of therapy delivered in one session per week;
- Combined family therapy and CBT for a total of 24 hours of therapy (two 1-hour sessions per week); and
- Psycho-education, which was delivered in eight 90-minute sessions for a total of 12 hours of therapy.

Following the 12-week treatment period, follow-up evaluations were conducted at 4 and 7 months. To determine outcome, the investigators assessed the number of days adolescents used marijuana, the proportion of adolescents who achieved minimal marijuana use (i.e., reported use on fewer than 10 percent of days), the results of urine drug screens, and the quantity and frequency of drug use.^3^

The adolescents who were assigned to the combined and psycho-education groups had shown the highest rates of marijuana use before treatment initiation, using the drug on 57 and 66 percent of days, respectively. The study found that these two groups also showed the greatest reduction in the proportion of days with marijuana use at the 7-month followup. However, these effects were specific to marijuana use because no significant changes were found in the number of days of alcohol or tobacco use in either of the groups.

### Study by Liddle and Colleagues (2001)

In a final study assessing the effectiveness of family therapy, 182 alcohol- and marijuana-abusing adolescents (ages 13–18) were randomly assigned to 14 to 16 weeks of (1) multidimensional family therapy, (2) adolescent group therapy, or (3) multifamily education intervention. Drug use, as determined by self-reports, collateral (i.e., parent or sibling) reports, or urine analysis, was used as the outcome measure at the end of the treatment period and at a 1-year follow-up assessment. The investigators found that adolescents assigned to multidimensional family therapy showed the greatest improvement (i.e., highest proportion of adolescents reporting reductions in drug use), with more than 40 percent of participants in this group reporting drug-use reduction. Moreover, this effect persisted throughout the 1-year follow-up period.

## Motivational Enhancement Therapy

Motivational enhancement therapy, or motivational interviewing (MI), is a brief intervention designed to enhance a person’s motivation to make changes regarding AOD use and those life situations that may trigger or sustain AOD use. (The same approach also can be used for other high-risk behaviors [Miller and Rollnick 2002].) To achieve this goal, MI uses an empathic, nonjudgmental approach; employs reflective listening; develops discrepancy (i.e., the therapist reflects back both the patient’s reasons to continue AOD use and the reasons to quit); avoids arguments; rolls with resistance (i.e., does not challenge the patient’s reasons for continuing AOD use and avoiding treatment); and supports the client’s own ability to change (i.e., self-efficacy for change). The MI approach is particularly appealing for treatment of AOD-abusing adolescents because these adolescents often do not seek treatment and need to be motivated to change their behaviors and seek treatment. Two studies have investigated the effectiveness of MI in AOD-abusing adolescents.

### Study by Marlatt and Colleagues (1998)

In this study, 188 female and 160 male high school seniors (age 19 or younger) were randomly assigned to a single brief MI session during their freshman year in college or to no intervention. The investigators then assessed drinking rates, alcohol-related problems, and alcohol dependence as determined by self-reports and reports by others on quantity and frequency of drinking as well as peak alcohol consumption; these outcomes were determined both at 6 months and at 2 years after the intervention.

The adolescents in the MI group exhibited significant reductions in both drinking and alcohol-related consequences at each of the follow-up points. Thus, at the 6-month followup they drank less frequently, had lower peak consumption levels, and lower consumption over time compared with the adolescents who did not receive an intervention. These effects also were seen at the 2-year followup, demonstrating that the benefits of MI can persist over time.

### Study by McCambridge and Strang (2004)

These investigators randomly assigned 200 adolescents (ages 16–20) to one MI session or to the control condition (i.e., education as usual without any intervention). The adolescents’ drug use at baseline was assessed using self-reports, peer interviews, and analysis of hair samples. Three months after the intervention, changes in the use of various drugs, drug-specific perceptions, and other behavioral outcomes were evaluated. The analysis found that overall the MI group showed significantly reduced nicotine, alcohol, and marijuana use. Moreover, the number of adolescents in the MI group who had been nondrinkers at baseline but had begun drinking at followup was significantly lower than the corresponding number of adolescents in the control group.

## Behavioral Therapy

Another psychosocial approach that has been used in the treatment of AOD-abusing adolescents is behavioral therapy, which targets AOD use in the context of the adolescent’s environment. In general, behavioral therapy attempts to identify the behaviors and situations in which AOD use occurs and then to disrupt those behaviors by equipping the client with skills to resist AOD use and avoid relapse. Consequently, one essential component of behavioral therapy is a functional analysis to explore what triggers AOD use and promotes continued use. Other components include skills training specific to each client (e.g., drug refusal skills and social skills) and relapse prevention, as well as stress management, assertiveness training, and self-regulation. One group of investigators has assessed the effectiveness of this approach in AOD-abusing adolescents, as described below.

### Study by Azrin and Colleagues (1994)

The study included 26 adolescents (ages 13–18, with a mean age of 16 years) who sought treatment for AOD use disorders. The participants were assigned either to behavioral therapy or supportive counseling. Components of the behavior therapy included the following:

- Written assignments and review of in-session assignments;
- Rehearsals of specific situations, with modeling by the therapist and self-recording;
- Procedures to identify the amount of time spent in “risky” versus “safe” situations, with the goal of increasing the time spent in safe situations;
- Urge control to disrupt internal stimuli that normally precede AOD use (e.g., thoughts of, and cravings for, alcohol); and
- Social control/contracting in which parents provided the adolescents with “safe” activities.

For the supportive counseling group, sessions focused on the expression of feelings, self-generated insight into the reasons for the adolescents’ AOD use, and discussion of drug-related experiences.

Both treatment approaches included an average of 15 sessions delivered over 6 months. Outcome was measured by determining type and frequency of drug use (based on both self-reports and urine tests), school attendance, employment, institutionalization, and arrest. The study found that adolescents in the behavioral therapy group reported less-frequent drug use (which was confirmed by fewer positive drug screens) and had improved school attendance and performance as well as better conduct ratings than adolescents in the supportive counseling group.

The same researchers also compared the effectiveness of behavioral therapy and supportive counseling in another group of 74 adolescents and adults (ages 13–43) who were followed for 9 months after therapy (Azrin et al. 1996). This analysis also demonstrated significantly greater reductions in AOD use in the behavioral therapy group than in the supportive therapy group, both at the end of treatment and at the 9-month followup. The behavioral therapy group also showed greater improvements in the number of days worked, days in school, and alcohol use.

Finally, the researchers further modified the behavioral therapy approach to include the adolescent’s family, an approach known as family behavioral therapy (Azrin et al. 2001). This intervention was compared with an individual cognitive problem-solving intervention in adolescents diagnosed with conduct disorder as well as AOD dependence. This analysis found that both interventions were equally effective.

## CBT

CBT, which, as described above, also has been combined with family therapy, is an extension of behavioral therapy that integrates cognitive aspects into behavioral strategies to address AOD use. Thus, CBT emphasizes functional analyses that help the client to better understand the factors and situations that precede AOD use as well as the consequences of that use. Accordingly, CBT focuses on identifying high-risk situations and helping the client acquire the skills necessary to prevent or appropriately address those situations. Two studies have investigated the effectiveness of this approach in adolescents.

### Study by Kaminer and Colleagues (1998)

This study included 32 adolescents (ages 13–18) who had been diagnosed with AOD abuse and other psychiatric disorders, such as disruptive disorders (e.g., conduct disorder or attention-deficit/hyperactivity disorder) or internalizing disorders (e.g., depression or an anxiety disorder). The participants were assigned to CBT or group therapy for 12 weeks. The CBT sessions included such components as presentations, modeling, role playing, and homework exercises. The outcomes measured included urine drug screen results, scores on a standardized assessment instrument (i.e., the Teen Addiction Severity Index), and self-reports on the quantity and frequency of AOD use.

The investigators initially hypothesized that adolescents with disruptive disorders might respond better to the CBT, whereas adolescents with internalizing disorders might achieve better outcomes with group therapy. However, the results did not support this hypothesis. Regardless of their coexisting disorder, adolescents in the CBT group achieved lower scores on the Teen Addiction Severity Index than did those receiving group therapy. Furthermore, no differences in treatment outcome existed between the groups with respect to the number of positive urine screens.

### Study by Kaminer and Colleagues (2002)

The same researchers later conducted a larger study comparing 88 adolescents (ages 13–18, with a mean age of 15.4 years) with AOD use disorders, most of whom also had been diagnosed with other psychiatric disorders. The participants received either CBT or psycho-educational therapy in 8 weekly 75- to 90-minute sessions. AOD use outcomes were determined by urine drug screens and scores on the Teen Addiction Severity Index, with follow-up assessments at 3 and 9 months.

Again, the investigators had formulated an initial hypothesis that although both groups would show improvements in drug use throughout the follow-up period, adolescents receiving CBT would exhibit better treatment retention and better outcomes at followup. The results, however, did not bear this out. Specifically, the researchers reported the following findings:

- Although the CBT group showed greater improvements at the 3-month followup, both groups had similar relapse rates at the 9-month followup—that is, the CBT group exhibited increasing relapse rates over time.
- At the 3-month followup, drug use, as determined by positive urine screens, was significantly lower among older adolescents and males receiving CBT than in the corresponding subgroups in the psycho-educational therapy group.
- Alcohol use decreased significantly up to the 3-month followup, particularly in the psycho-educational therapy group; conversely, other drug use over those 3 months declined more in the CBT group.
- Improvements in self-reported AOD use were similar in both groups between the 3-month and 9-month followups.

## Pharmacotherapy

Several medications have been approved by the U.S. Food and Drug Administration for the treatment of alcohol dependence in adults, including disulfiram (Antabuse^®^), naltrexone (ReVia^®^), and acamprosate (Campral^®^). However, these and other agents are rarely used in the treatment of adolescents with AOD use disorders and have not been studied in this population. Only two double-blind, placebo-controlled trials^4^ have assessed the use of pharmacotherapy in adolescents with AOD use disorders, and the medications tested were for the treatment of coexisting psychiatric disorders rather than for AOD use disorders.

### Study by Geller and Colleagues (1998)

These investigators randomly assigned 25 adolescents (ages 12–18, with a mean age of 16.3 years) with bipolar disorder and a resulting AOD use disorder to treatment with lithium (a mood stabilizer that counteracts both mania and depression) or placebo for 6 weeks. In addition, all participants received weekly interpersonal therapy and were seen twice weekly by a health care provider. Outcome measures included AOD use as determined by urine drug screens and clinical improvement as determined by a standardized instrument (i.e., the Clinical Global Assessment Scale). The study found that adolescents in the lithium group had significantly fewer positive drug screens and exhibited greater clinical improvement; however, mood outcomes did not differ between the two groups.

### Study by Deas and Colleagues (2000a)

In this study, 10 adolescents (mean age 16.8 years) with alcohol dependence and co-occurring depression were randomly assigned to 12 weeks of treatment with the antidepressant sertraline or a placebo. In addition, all participants received CBT during those 12 weeks. To determine outcome, quantity and frequency of alcohol use and changes in depression scores were assessed and comparable reductions were found in both groups. The lack of difference between the two groups may result from the fact that both groups received CBT, which has been proven to be effective in the treatment of AOD use disorders. In addition, the number of participants was so small that differences between the groups may not have become apparent; a larger sample size might have been able to distinguish between the effects of the therapy and any additional medication effects.

## Conclusions

As the studies reviewed here demonstrate, much progress has been made in the development and implementation of treatment approaches for adolescents with AOD use problems as well as in the analysis of their effectiveness. Moreover, the analyses demonstrate that various treatment approaches can result in beneficial outcomes for the adolescents. Also noteworthy is the fact that most of these studies assessed and targeted multiple drugs of abuse because adolescents tend to use not just one drug. For example, the two most commonly used drugs among adolescents—alcohol and marijuana—frequently are used together.

Nevertheless, these studies are associated with a number of limitations. First, few studies addressed the adolescents’ developmental status and how it may affect treatment outcome, primarily because the age ranges included were so broad that developmental issues were difficult to address. And even when cognitive development was considered in the context of MST and CBT treatment approaches, no conclusive statements could be made regarding the impact of cognitive development on outcome because there was too much variance among the participants. Second, the outcomes assessed and the methods to determine AOD use and other outcomes differed greatly among the studies, making direct comparisons impossible. Third, most studies compared the treatment approach under investigation with other approaches but did not include a no-treatment control group. Although it clearly would be unethical to withhold treatment from adolescents with AOD use disorders, the lack of such control groups somewhat limits interpretation of the study results. Thus, despite the progress in research on AOD treatment for adolescents, future studies can be improved—for example, by using state-of-the-art assessment instruments designed specifically for adolescents and by more consistently integrating assessments of the adolescents’ developmental status and its impact on treatment outcome.

## Footnotes

1 The rate of use of hard drugs was too low to allow for statistical analyses.

2 This approach focuses on treatment engagement, addressing inappropriate alliances, and identifying maladaptive interactions to create healthier relationships.

3 Drug use quantity and frequency was determined based on self-reports as well as on reports by parents and siblings.

4 The term “double-blind” means that neither the patient nor the administering physician know if the patient gets the active medication or an inactive “dummy-pill” (i.e., placebo).

## References

[b1-arh-32-1-76] Azrin NH, Acierno R, Kogan ES (1996). Follow-up results of supportive versus behavioral therapy for illicit drug use. Behavior Research & Therapy.

[b2-arh-32-1-76] Azrin NH, Donohue B, Besalel V (1994). Youth drug abuse treatment: A controlled outcome study. Journal of Child and Adolescent Substance Abuse.

[b3-arh-32-1-76] Azrin NH, Donohue B, Teichner G (2001). A controlled evaluation and description of individual-cognitive problem solving and family-behavior therapies in dually diagnosed conduct-disordered and substance-dependence youth. Journal of Child and Adolescent Substance Abuse.

[b4-arh-32-1-76] Deas D (2008). Evidence-based treatments for alcohol use disorders in adolescents. Pediatrics.

[b5-arh-32-1-76] Deas D, Randall CL, Roberts JS, Anton RF (2000a). A double-blind, placebo-controlled trial of sertraline in depressed adolescent alcoholics: A pilot study. Human Psychopharmacology.

[b6-arh-32-1-76] Deas D, Riggs P, Langenbucher J (2000b). Adolescents are not adults: Developmental considerations in alcohol users. Alcoholism: Clinical and Experimental Research.

[b7-arh-32-1-76] Geller B, Cooper TB, Sun K (1998). Double-blind and placebo-controlled study of lithium for adolescent bipolar disorders with secondary substance dependence. Journal of the American Academy of Child and Adolescent Psychiatry.

[b8-arh-32-1-76] Henggeler S, Bourdin C, Melton G (1991). Effects of multisystemic therapy on drug use and abuse in serious juvenile offenders: A progress report from two outcome studies. Family Dynamics and Addiction Quarterly.

[b9-arh-32-1-76] Henggeler SW, Clingempeel WG, Brondino MJ, Pickrel SG (2002). Four-year follow-up of multisystemic therapy with substance-abusing and substance-dependent juvenile offenders. Journal of the American Academy of Child and Adolescent Psychiatry.

[b10-arh-32-1-76] Henggeler SW, Pickrel SG, Brondino MJ (1999). Multisystemic treatment of substance-abusing and dependent delinquents: Outcomes, treatment fidelity, and transportability. Mental Health Services Research.

[b11-arh-32-1-76] Joanning H, Quinn Q, Thomas F, Mullen R (1992). Treating adolescent drug abuse: A comparison of family systems therapy, group therapy, and family drug education. Journal of Marital and Family Therapy.

[b12-arh-32-1-76] Kaminer Y, Burleson JA, Goldberger R (2002). Cognitive–behavioral coping skills and psychoeducation therapies for adolescent substance abuse. Journal of Nervous and Mental Disease.

[b13-arh-32-1-76] Kaminer Y, Burleson JA, Blitz C (1998). Psychotherapies for adolescent substance abusers: A pilot study. Journal of Nervous and Mental Disease.

[b14-arh-32-1-76] Latimer WW, Winters KC, D’Zurilla T, Nichols M (2003). Integrated family and cognitive–behavioral therapy for adolescent substance abusers: A stage I efficacy study. Drug and Alcohol Dependence.

[b15-arh-32-1-76] Lewis RA, Piercy FP, Sprenkle DH, Trepper TS (1990). Family-based interventions for helping drug-abusing adolescents. Journal of Adolescent Research.

[b16-arh-32-1-76] Liddle HA, Dakof GA, Parker K (2001). Multidimensional family therapy for adolescent drug abuse: Results of a randomized clinical trial. American Journal of Drug and Alcohol Abuse.

[b17-arh-32-1-76] Marlatt GA, Baer JS, Kivlahan DR (1998). Screening and brief intervention for high-risk college student drinkers: Results from a 2-year follow-up assessment. Journal of Consulting and Clinical Psychology.

[b18-arh-32-1-76] McCambridge J, Strang J (2004). The efficacy of single-session motivational interviewing in reducing drug consumption and perceptions of drug-related risk and harm among young people: Results from a multi-site cluster randomized trial. Addiction.

[b19-arh-32-1-76] Miller WR, Rollnick S (2002). Motivational Interviewing: Preparing People for Change.

[b20-arh-32-1-76] Waldron HB, Slesnick N, Brody JL (2001). Treatment outcomes for adolescent substance abuse at 4- and 7-month assessments. Journal of Consulting and Clinical Psychology.

